# Comprehensive analysis of TLX2 in pan cancer as a prognostic and immunologic biomarker and validation in ovarian cancer

**DOI:** 10.1038/s41598-023-42171-5

**Published:** 2023-09-27

**Authors:** Buze Chen, Xiaojuan Ding, Ailing Wan, Xin Qi, Xiaoman Lin, Haihong Wang, Wenyu Mu, Gang Wang, Junnian Zheng

**Affiliations:** 1 Xuzhou Medical University, Xuzhou, 221004 Jiangsu China; 2 Cancer Institute, Xuzhou Medical University, No. 209 Tongshan Road, Yunlong District, Xuzhou, 221004 Jiangsu China; 3 Department of Gynecology, The Affiliated Hospital of Xuzhou Medical University, No. 99 West Huaihai Road, Quanshan District, Xuzhou, 221002 Jiangsu China; 4 Center of Clinical Oncology, The Affiliated Hospital of Xuzhou Medical University, No. 99 West Huaihai Road, Quanshan District, Xuzhou, 221002 Jiangsu China; 5 Jiangsu Center for the Collaboration and Innovation of Cancer Biotherapy, Xuzhou Medical University, Xuzhou, 221004 Jiangsu China

**Keywords:** Biomarkers, Oncology

## Abstract

T cell leukemia homeobox 2 (*TLX2*) plays an important role in some tumors. Bioinformatics and experimental validation represent a useful way to explore the mechanisms and functions of *TLX2* gene in the cancer disease process from a pan cancer perspective. *TLX2* was aberrantly expressed in pan cancer and cell lines and correlated with clinical stage. High *TLX2* expression was significantly associated with poor overall survival in COAD, KIRC, OC, and UCS. The greatest frequency of *TLX2* alterations in pan cancer was amplification. Alterations of *NXF2B*, *MSLNL*, *PCGF1*, *INO80B-WBP1*, *LBX2-AS1*, *MRPL53*, *LBX2*, *TTC31*, *WDR54*, and *WBP1* co-occurred in the *TLX2* alteration group. PFS was significantly shorter in the *TLX2*-altered group (n = 6) compared to the *TLX2*-unaltered group (n = 400). Methylation levels of TLX2 were high in 17 tumors. *TLX2* expression was associated with MSI in seven tumors and TMB in five tumors. *TLX2* expression was associated with immune infiltration and immune checkpoint genes. *TLX2* may be associated with some pathways and chemoresistance. We constructed a possible competing endogenous RNA (ceRNA) network of *LINC01010*/miR-146a-5p/*TLX2* in OC. *TLX2* expression was significantly upregulated in ovarian cancer cell lines compared to ovarian epithelial cell lines. Aberrant expression of *TLX2* in pan cancer may promote tumorigenesis and progression through different mechanisms. *TLX2* may represent an important therapeutic target for human cancers.

## Introduction

Globally, cancer is one of the most common causes of death, with cancer set to kill nearly 10 million people by 2020^1,2^. Given the morbidity and mortality of cancer, there is a need to find new biomarkers to diagnose and predict the prognosis of various tumors and provide new strategies for the treatment of tumors.

T-cell leukemia homologous frame 2 (*TLX2*) was found to be a member of the *HOX11* homologous frame gene family^3^, also known as *NCX*^4^, *HOX11L1*^5^, and *ENX*^6^. It encodes a protein consisting of 285 amino acids. Allelic deletion at the *TLX2* locus predicts the outcome of gastrointestinal mesenchymal tumors^7^. TLX2 may mediate the tumorigenesis of canine malignant melanoma^8^. *TLX2* is an mRNA stemness index (mRNAsi)-related differentially expressed gene in lung squamous carcinoma (LUSC)^9^. A novel 6-gene (*FGF23*, *TLX2*, *TIFAB*, *RNF223*, *HIST1H3A*, and *AADACL4*) signature improves prognostic prediction in patients with endometrial cancer^10^. However, the role of *TLX2* in pan cancer remains unknown.

In recent years, the use of immunotherapy as a modern cancer treatment has been expanding in clinical applications^11^. Anticancer immunotherapy has encountered its own opportunities, with several reports identifying biomarkers for immune checkpoint inhibitors (ICIs), such as immune checkpoint gene expression and tumor mutation burden (TMB)^12^. Immunotherapy has rapidly become a cornerstone of many cancer therapies, but the response to immunotherapy has not been very promising, with only some patients showing a durable response to immunotherapy^13^. Therefore, there is a need to better understand the mechanisms of immunotherapy against cancer.

Pan cancer analysis is useful to help us initially explore the function of genes. This study reveals the potential mechanism of *TLX2* in pan cancer based on data obtained from various datasets. In this study, we performed *TLX2* analysis in pan cancer to investigate the association between *TLX2* expression and prognosis, *TLX2* expression and staging, the relationship between microsatellite instability (MSI) and tumor mutational load (TMB), genetic variation analysis of *TLX2*, the relationship between *TLX2* expression and immune infiltration, the relationship between *TLX2* expression and immune checkpoint genes (ICG), possible regulatory networks of *TLX2*, TLX2-mediated drug sensitization, *TLX2* expression pattern in single cells and its relationship with functional status of the cancer.

## Materials and methods

### Expression of TLX2 in pan cancer and cell lines

R programming language (Version 3.6.3) was used for statistical analysis and visualization. The package ggplot2 [version 3.3.3] was used for visualization. The molecule was *TLX2* [ENSG00000115297]. We processed UCSC XENA (https://xenabrowser.net/datapages/, accessed on 10 June 2022) into RNAseq data in TPM format for TCGA (https://portal.gdc.cancer.gov, accessed on 10 June 2022) and GTEx by Toil process^14–18^. We log-transformed the RNAseq data for comparative analysis. Data were not filtered for subgroups. Subgroups included ACC (adrenocortical carcinoma), BLCA (bladder urothelial carcinoma), BRCA (breast invasive carcinoma), CESC (cervical squamous cell carcinoma and endocervical adenocarcinoma), CHOL (cholangiocarcinoma), COAD (colon adenocarcinoma), DLBC (lymphoid neoplasm diffuse large B-cell lymphoma), ESCA (esophageal carcinoma), GBM (glioblastoma multiforme), HNSC (head and neck squamous cell carcinoma), KICH (kidney chromophobe), KIRC (kidney renal clear cell carcinoma), KIRP (kidney renal papillary cell carcinoma), LAML (acute myeloid leukemia), LGG (brain lower grade glioma), LIHC (liver hepatocellular carcinoma), LUAD (lung adenocarcinoma), LUSC (lung squamous cell carcinoma), MESO (mesothelioma), OC (ovarian serous cystadenocarcinoma), PAAD (pancreatic adenocarcinoma), PCPG (pheochromocytoma and paraganglioma), PRAD (prostate adenocarcinoma), READ (rectum adenocarcinoma), SARC (sarcoma), SKCM (skin cutaneous melanoma), STAD (stomach adenocarcinoma), TGCT (testicular germ cell tumors), THCA (thyroid carcinoma), THYM (thymoma), UCEC (uterine corpus endometrial carcinoma), UCS (uterine carcinosarcoma), and UVM (uveal melanoma)^18,19^. The statistical method was Wilcoxon rank sum test.

We analyzed TLX2 expression in human cancer cell lines (n = 641) using the Cancer Cell Line Encyclopedia (CCLE) database (https://www.broadinstitute.org/ccle, accessed on 17 August 2023)^20,21^.

### Correlation analysis of TLX2 and prognosis in pan cancer

We used univariate Cox regression and the package forestplot for the analyses. We analyzed overall survival (OS), progression-free survival (PFS), and disease-specific survival (DSS). The statistical method was Wilcox test.

### Diagnostic value of TLX2 in pan cancer

We used the package pROC [1.18.0] to perform ROC analysis on the data and the results were visualized using package ggplot2 [3.3.6]. The data processing method is log2 (value + 1).

If a specific tumor contains normal samples in TCGA, the data from TCGA were used. If the specific tumor has no normal samples in TCGA, data from TCGA + GTEx were used.

### Correlation between TLX2 expression and stage, TMB, and MSI in pan cancer

We used the GEPIA 2 website (http://gepia2.cancer-pku.cn/#index, accessed on 10 June 2022) to analyze the expression of *TLX2* at different clinical stages in pan cancer^18^. We used the “Expression DIY” module to analyze the expression of a gene in different cancer stages using the box plot.

We obtained RNAseq data (level3) and corresponding clinical information from the TCGA database for 33 tumors. We obtained TMB and MSI from the literature^22,23^. The statistical method was Wilcox test.

### Genomic alterations of TLX2 in pan cancer

The cBioPortal database (http://www.cbioportal.org/, accessed on 10 June 2022) was used to analyze the genetic alterations of *TLX2* in the TCGA pan cancer dataset^24^. We analyzed *TLX2* genetic alterations and mutant sites based on the “Oncoprint”, “Cancer Type Summary”, and “Mutation” modules^18^. The statistical method was Logrank test.

We analyzed the methylation level of TLX2 in pan cancer using MethHC 2.0 (http://awi.cuhk.edu.cn/∼MethHC, accessed on 17 August 2023)^25,26^. We used the “GENE METHYLATION” module for the analysis.

### Correlation of TLX2 expression with immune infiltration and immune checkpoint genes

We explored the association between *TLX2* expression and cancer-associated fibroblast (CAF) infiltration using the “immune” module of TIMER2 (http://timer.cistrome.org/, accessed on 10 June 2022). Algorithms included Extended Multidimensional Immunome Characterization (EPIC), Microenvironmental Cell Population-Counter (MCP-counter), Cell Type Enrichment Analysis (XCELL) and Tumor Immune Dysfunction and Exclusion (TIDE) algorithms^18^. The data were analyzed using Spearman’s correlation analysis.

The immune checkpoint genes including *SIGLEC15*, *IDO1*, *CD274*, *HAVCR2*, *PDCD1*, *CTLA4*, *LAG3*, and *PDCD1LG2* were obtained from the literature^18^. We extracted the expression values of these eight genes. The statistical method was Wilcox test.

### TLX2-related functional enrichment analysis

We analyzed proteins for *TLX2* interactions using the STRING website (version 11.5, https://string-db.org/, accessed on 10 June 2022). The confidence score was 0.4^27^. We used “Similar Genes Detection” module of GEPIA2 to analyze the top 100 genes with similar expression patterns to TLX2 in pan cancer. We searched for genes with similar expression patterns to TLX2 genes in pan cancer.

We performed GO enrichment analysis and KEGG pathway analysis of *TLX2* and interacting proteins using the DAVID website (https://david.ncifcrf.gov/, accessed on 10 June 2022)^27^.

### Drug sensitivity of TLX2 in pan cancer

We used the RNAactDrug database (http://bio-bigdata.hrbmu.edu.cn/RNAactDrug/index.jsp, accessed on 10 June 2022) to analyze the drug sensitivity of *TLX2* in pan cancer^18^. We selected the top 5 significantly positively correlated drugs and the top 5 significantly negatively correlated drugs based on Spearman’s correlation coefficient^18^.

### Single cell sequencing data analysis

We used the CancerSEA database (http://biocc.hrbmu.edu.cn/CancerSEA/home.jsp, accessed on 10 June 2022) on the server to explore the correlation between *TLX2* expression and different tumor functional states. It had only data for GBM and RB. The plot showing the correlation between TLX2 expression and different functional status in GBM was obtained from the CancerSEA database. We obtained t-SNE plots of individual cells from the CancerSEA database.

### CeRNA network construction

We focused on the mining of molecular markers in OC. To explore the possible competing endogenous RNA (ceRNA) network of TLX2 in OC, we used TarBase V.8 (https://carolina.imis.athena-innovation.gr/diana_tools/web/index.php?r=tarbasev8%2Findex, accessed on 10 June 2022) to predict the binding of TLX2-binding miRNAs^17,28^. We screened miRNAs based on their expression in OC and correlation with OC prognosis. Next, we used LncBase Predicted v.2 (https://carolina.imis.athena-innovation.gr/diana_tools/web/index.php?r=lncbasev2/index-predicted, accessed on 10 June 2022) to predict the lncRNAs that interact with miRNAs bound to lncRNAs. We screened lncRNAs based on their expression in OC and correlation with OC prognosis.

We used GSE103708 to analyze the expression of miR-146a-5p in normal ovarian and OC tissues^24,29^. The molecules were *LINC01010* [ENSG00000236700.6] and *TLX2*. The statistical method was the t-test.

We used R programming language (version 3.6.3), package survminer [version 0.4.9], and package survival [version 3.2-10] for statistical analysis and visualization^15^. Molecules were hsa-miR-146a-5p [MIMAT0000449], *LINC01010*, and *TLX2*. Subgroups were 0–50 vs 50–100 (median threshold of expression)^30^. The prognostic type was OS. We used miRNAseq data from the TCGA-OC project for level 3 BCGSC miRNA profiles and RNAseq data from the TCGA-OC project for level 3 HTSeq-FPKM format^17,27^. Prognostic data were obtained from the literature^31^. The statistical method was the Welch t test.

### QRT-PCR

The human ovarian surface epithelial cell line OSE29, OC cell lines SKOV3 and A2780 were preserved in our laboratory. qRT-PCR was used to detect the expression levels of *TLX2* in OSE29, SKOV3, and A2780 cell lines. The primer sequences used are as follows. *GAPDH*, Forward: CGACAGTCAGCCGCATCTTC, Reverse: CGTTCTCAGCCTTGACGGTG; *TLX2*, Forward: 5′-GGTTCTCCTCGGCCCAGA-3′, Reverse: 5′-GCCGATCGGACGGGCGT-3′^18,32^. The statistical method was the t-test.

### Statistical analysis

We performed statistical analysis using R programming language (version 3.6.3). P-values below 0.05 were considered statistically significant.

## Results

### Abnormal expression of TLX2 in pan cancer and cell lines

**Figure 1:**
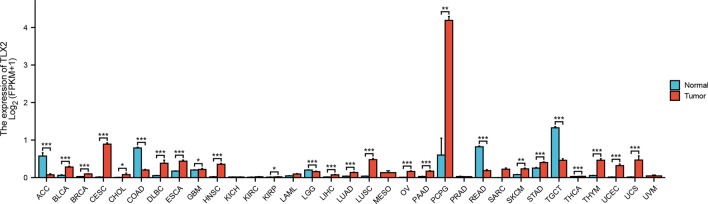
Differential *TLX2* expression between tumor and normal samples acquired from TCGA and GTEx. Red columns represent cancer samples, blue columns represent normal samples, *P < 0.05; **P < 0.01; ***P < 0.001.

As shown in Fig. 1, *TLX2* expression was significantly upregulated in BLCA, BRCA, CESC, CHOL, DLBC, ESCA, HNSC, KIRC, LAML, LIHC, LUAD, LUSC, OC, PCPG, PRAD, STAD, UCEC, and UCS, and significantly downregulated in ACC, COAD, GBM, LGG, READ, and TGCT compared with normal tissues. Abnormal *TLX2* expression in pan cancer may be associated with the development and progression of these tumors. As shown in Fig. S1, analysis of the CCLE dataset also showed differential expression of some cell lines.

### TLX2 expression correlates with prognosis in pan cancer

**Figure 2:**
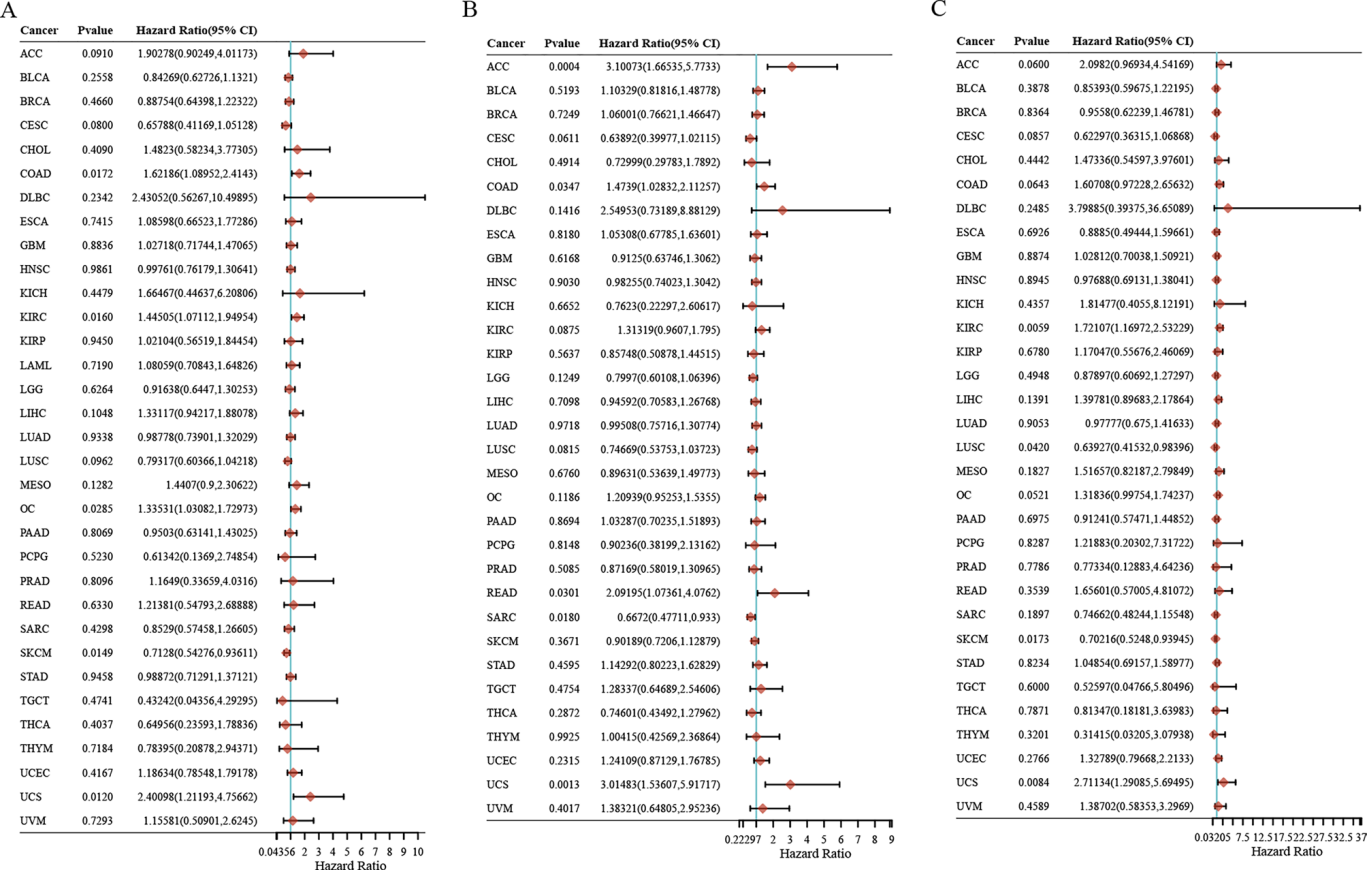
Expression of *TLX2* in pan cancer correlates with prognosis. (**A**) OS, (**B**) PFS, (**C**) DSS.

As shown in Fig. 2A, *TLX2* high expression showed poor OS of COAD (HR 1.62; 95% CI 1.09–2.41; P = 0.0172), KIRC (HR 1.45; 95% CI 1.07–1.95; P = 0.016), OC (HR 1.34; 95% CI 1.03–1.73; P = 0.029), and UCS (HR 2.40; 95% CI 1.21–4.76; P = 0.012). Low expression of *TLX2* was significantly associated with a better OS in SKCM (HR 0.71; 95% CI 0.54–0.94; P = 0.015). As shown in Fig. 2B, *TLX2* high expression showed poor PFS of ACC (HR 3.10; 95% CI 1.67–5.77; P = 0.0004), COAD (HR 1.47; 95% CI 1.03–2.11; P = 0.035), READ (HR 2.09; 95% CI 1.07–4.08; P = 0.03), and UCS (HR 3.01; 95% CI 1.54–5.92; P = 0.001). Low expression of *TLX2* was significantly associated with a better PFS in SARC (HR 0.67; 95% CI 0.48–0.93; P = 0.018). As shown in Fig. 2C, *TLX2* high expression showed poor DSS of KIRC (HR 1.72; 95% CI 1.17–2.53; P = 0.006) and UCS (HR 2.71; 95% CI 1.29–5.69; P = 0.0084). Low expression of *TLX2* was significantly associated with a better DSS in LUSC (HR 0.64; 95% CI 0.42–0.98; P = 0.042) and SKCM (HR 0.70; 95% CI 0.52–0.94; P = 0.017).

### The diagnostic value of TLX2 in tumors

**Figure 3:**
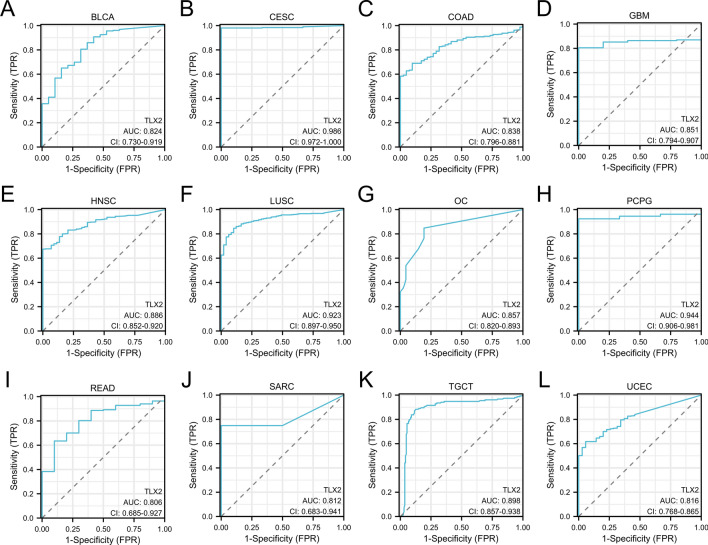
ROC curves showed that *TLX2* had a high diagnostic value (AUC > 0.8) in several types of cancers. (**A**) BLCA, (**B**) CESC, (**C**) COAD, (**D**) GBM, (**E**) HNSC, (**F**) LUSC, (**G**) OC, (**H**) PCPG, (**I**) READ, (**J**) SARC, (**K**) TGCT, (**L**) UCEC.

ROC curves were depicted to explore the diagnostic value of *TLX2* in 33 tumors. The ROC curves showed that the *TLX2* gene was found in BLCA (AUC = 0.824), CESC (AUC = 0.986), COAD (AUC = 0.838), GBM (AUC = 0.851), HNSC (AUC = 0.886), LUSC (AUC = 0.923), OC (AUC = 0.857), PCPG (AUC = 0.944), OC (AUC = 0.972), READ (AUC = 0.806), SARC (AUC = 0.812), TCGT (AUC = 0.898), and UCEC (AUC = 0.816) (Fig. 3).

### TLX2 expression between different clinical characteristics

**Figure 4:**
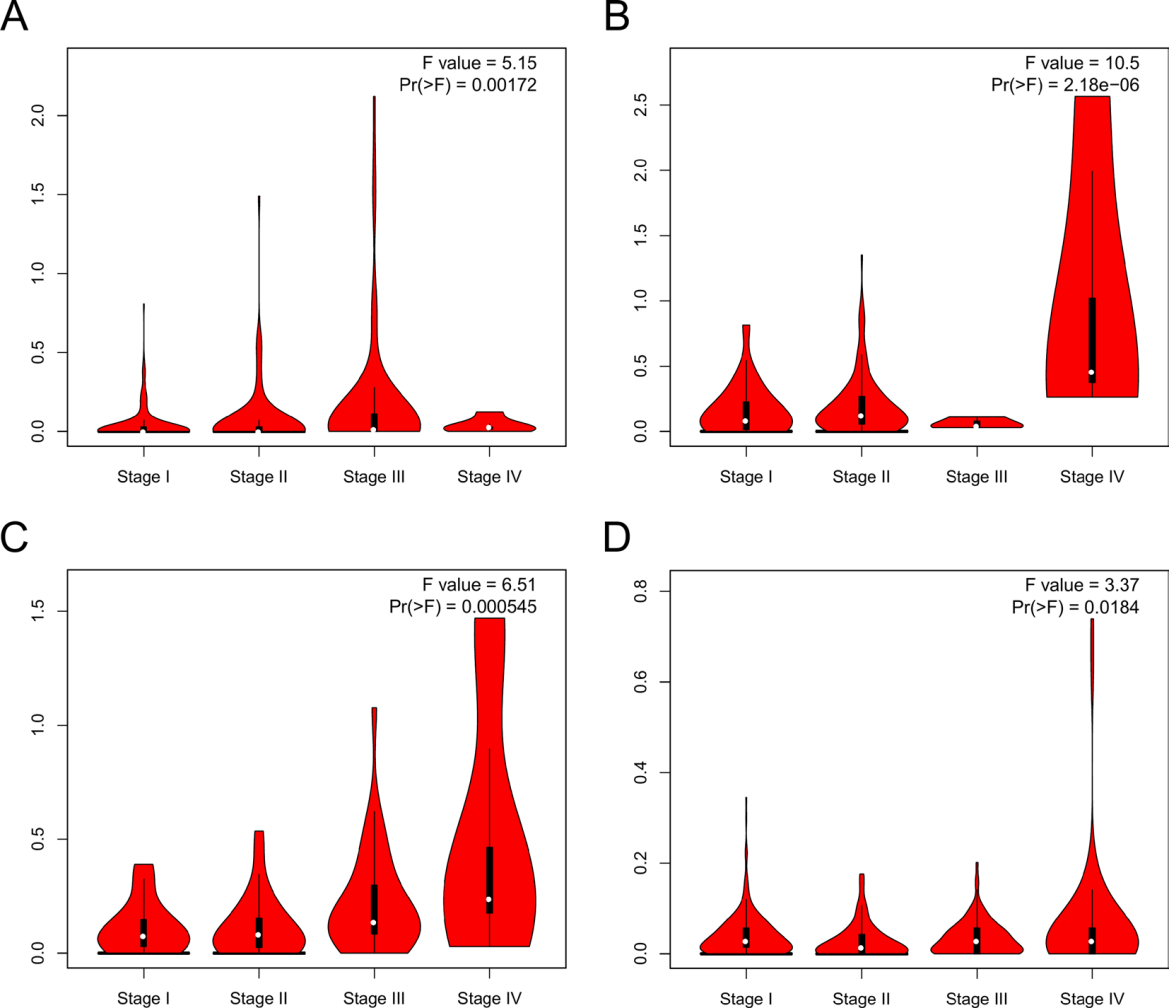
*TLX2* expression in pan cancer correlated with pathological stage. (**A**) LIHC, (**B**) PAAD, (**C**) READ, (**D**) THCA.

**Figure 5:**
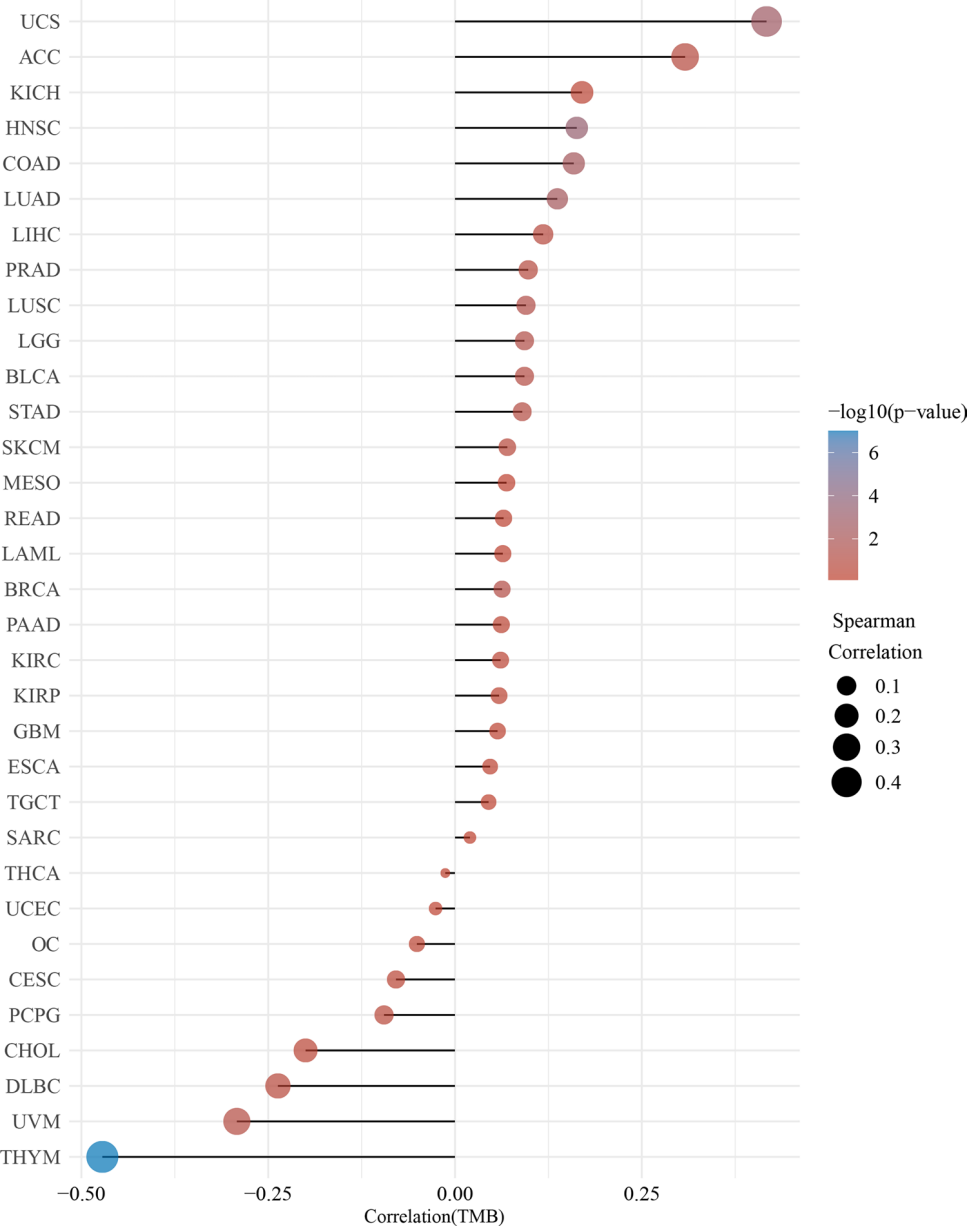
The expression of *TLX2* in pan cancer was correlated with tumor mutational burden (TMB).

**Figure 6:**
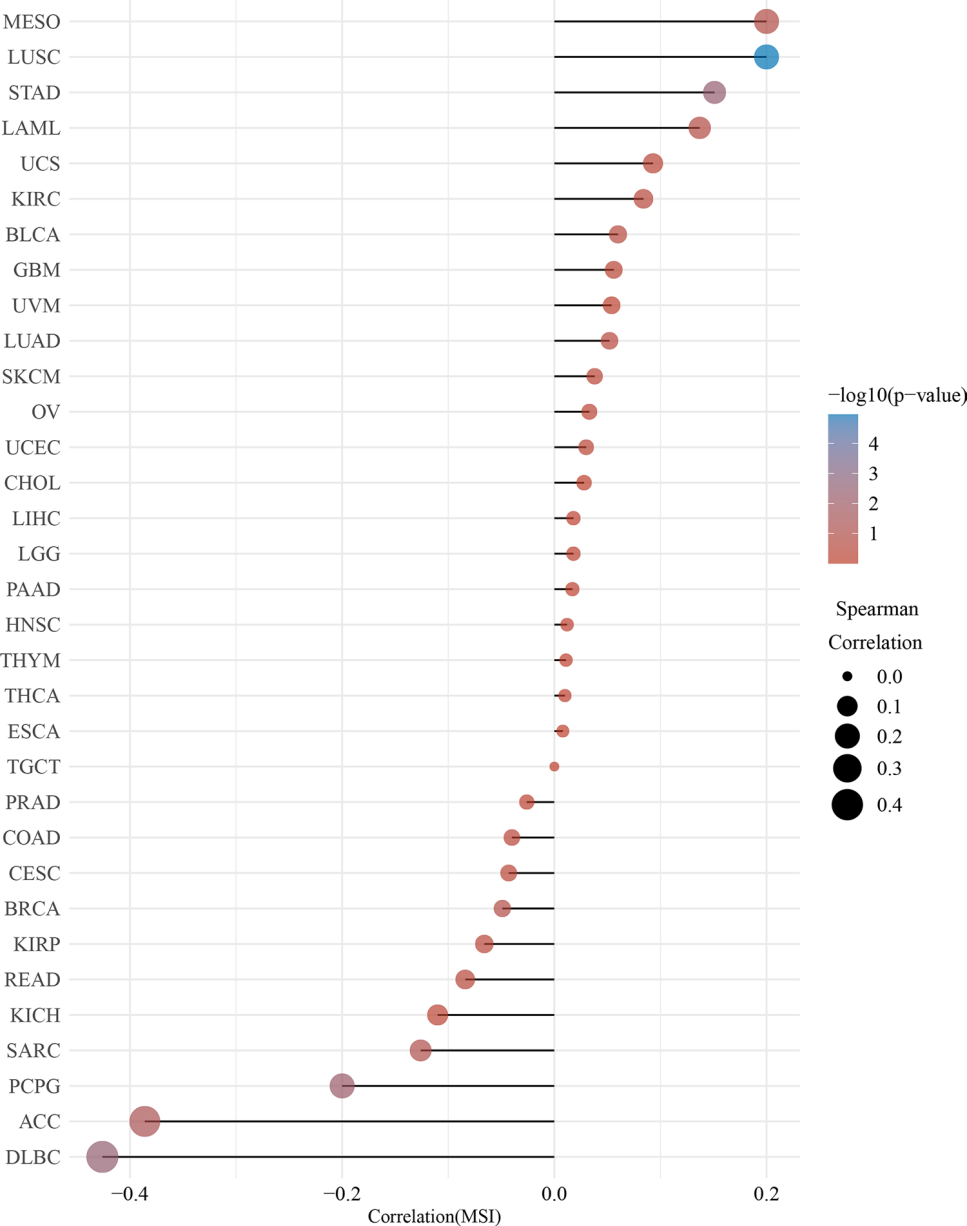
The expression of *TLX2* in pan cancer was correlated with microsatellite instability (MSI).

As shown in Fig. 4, high *TLX2* expression correlated with the stage of LIHC, PAAD, READ, and THCA, and not with the stage of the other 29 tumors. As shown in Fig. 5, *TLX2* expression was positively correlated with MSI status of LUSC (P < 0.0001) and STAD (P = 0.0073), while negatively correlated with MSI status of ACC (P = 0.0431), DLBC (P = 0.0035), and PCPG (P = 0.0073). As shown in Fig. 6, *TLX2* expression was positively correlated with the TMB status of COAD (P = 0.0040), HNSC (P = 0.0004), LGG (P = 0.0464), LUAD (P = 0.0042), LUSC (P = 0.0444), and UCS (P = 0.0015), and negatively correlated with the TMB status of THYM (P < 0.0001).

### Genetic alterations and methylation levels of TLX2 in different tumors

**Figure 7:**
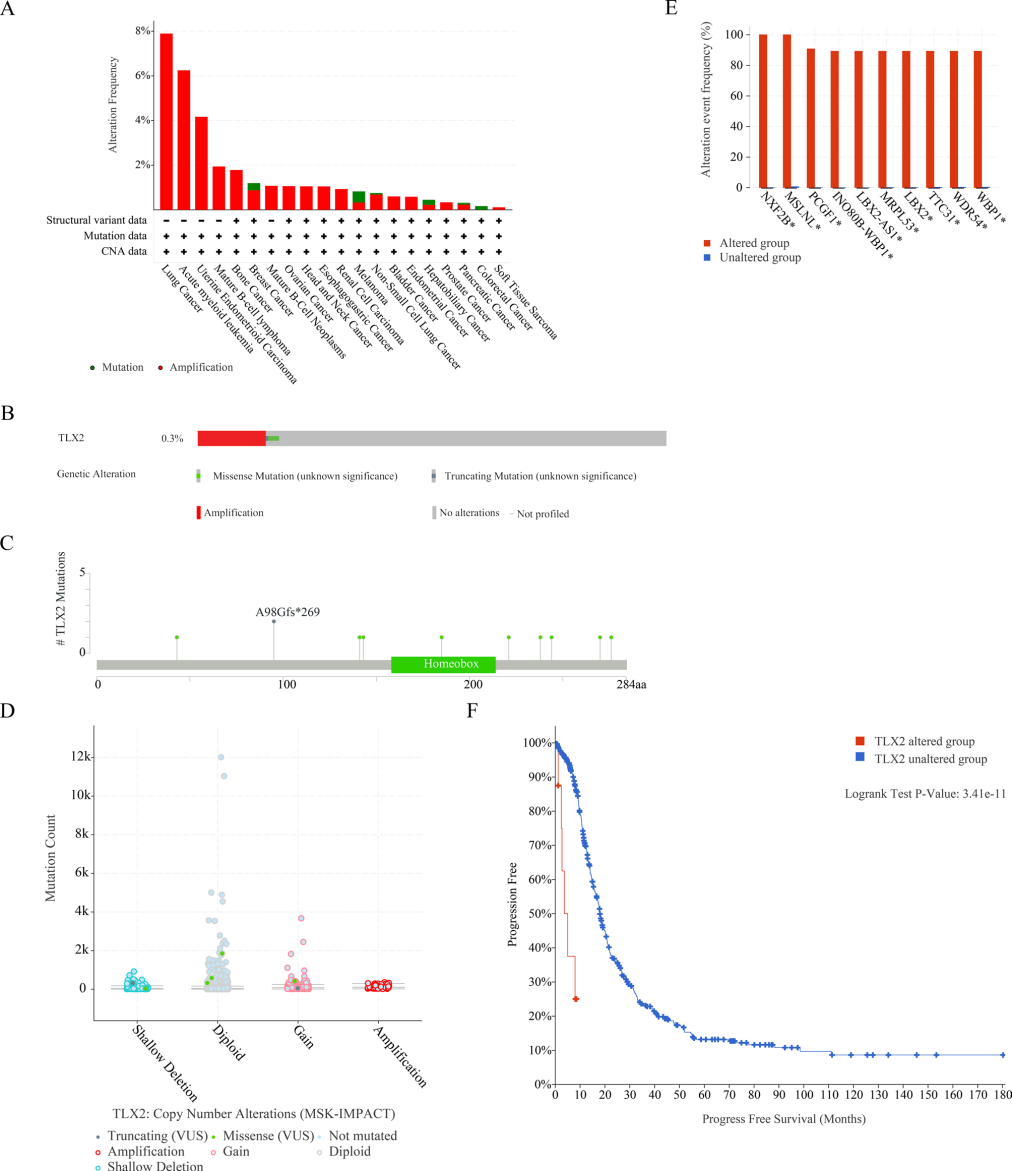
Genomic alterations of *TLX2* in pan cancer. (**A**) Summary of *TLX2* alterations in pan cancer. (**B**) Structural variants, mutations, and copy number alterations of *TLX2*. (**C**) Mutation types, number, and loci of *TLX2* gene alterations. (**D**) Types of *TLX2* alterations in pan cancer. (**E**) Top 10 genes co-altered with *TLX2* alterations. (**F**) The group of *TLX2* alterations in OC suggested poor prognosis.

As shown in Fig. 7A, there were variants of *TLX2* in pan cancer, including lung cancer (amplification 7.89%), acute myeloid leukemia (amplification 6.25%), uterine endometrioid carcinoma (amplification 4.17%), mature B-cell lymphoma (amplification 1.94%), bone cancer (amplification 1.79%), breast cancer (amplification 0.98%; mutation 0.32%), mature B-Cell neoplasms (amplification 1.08%), ovarian cancer (amplification 1.06%), head and neck cancer (amplification 1.05%), esophagogastric cancer (amplification 1.05%), renal cell carcinoma (amplification 0.93%), melanoma (amplification 0.33%; mutation 0.5%), non-small cell lung cancer (amplification 0.68%; mutation 0.07%), bladder cancer (amplification 0.6%), endometrial cancer (amplification 0.58%), hepatobiliary cancer, prostate cancer (amplification 0.34%), pancreatic cancer (amplification 0.23%; mutation 0.08%), colorectal cancer (mutation 0.17%), and soft tissue sarcoma (amplification 0.11%). As illustrated in Fig. 7B, the variation frequency of *TLX2* was 0.3%, and the variation types included amplification, missense mutation, and truncation mutation. As shown in Fig. 7C, the *TLX2* variant sites included 9 missense mutations (G43D, G141V, R143C, A185V, E221K, A238T, R244W, S276L, E270K) and 2 truncating mutations (A98Gfs*269 gain; A98Gfs*269 shallow Del). As illustrated in Fig. 7D, the copy number alterations of *TLX2* include amplification, gain, diploid, and shallow deletion. Gene alterations in *NXF2B*, *MSLNL*, *PCGF1*, *INO80B-WBP1*, *LBX2-AS1*, *MRPL53*, *LBX2*, *TTC31*, *WDR54*, and *WBP1* were more common in the altered group than in the unaltered group (Fig. 7E). As illustrated in Fig. 7F, PFS was significantly lower in the *TLX2* altered group (n = 6) than in the *TLX2* unaltered group (n = 400) in OC patients (P = 3.41e−11). As shown in Table S1, methylation levels of TLX2 were high in 17 tumors, including BLCA, BRCA, CESC, CHOL, COAD, ESCA, HNSC, KIRC, KIRP, LIHC, LUAD, LUSC, PAAD, PRAD, READ, THCA, and UCEC.

### Expression of TLX2 in pan cancer was associated with immune infiltration and immune checkpoint genes

**Figure 8:**
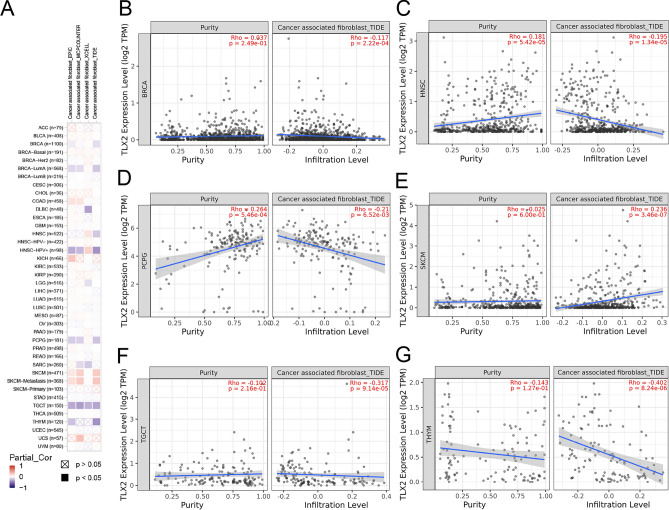
*TLX2* expression in pan cancer correlates with cancer-associated fibroblasts (CAF). (**A**) TIMER2.0 for *TLX2* expression and CAF correlation. (**B**) BRCA, (**C**) HNSC, (**D**) PCPG, (**E**) SKCM, (**F**) TGCT, (**G**) THYM.

As shown in Fig. 8A, the expression of *TLX2* in some tumors (COAD, KICH, SKCM, and UCS) was positively correlated with CAF, while the expression of *TLX2* in some tumors (BRCA, DLBC, LGG, PCPG, SARC, TGCT, and THYM) was negatively correlated with CAF. The TIDE algorithm showed that *TLX2* expression in BRCA (Fig. 8B), HNSC (Fig. 8C), PCPG (Fig. 8D), TGCT (Fig. 8F), and THYM (Fig. 8G) was significantly negatively correlated with CAF, while *TLX2* expression in SKCM (Fig. 8E) was significantly positively correlated with CAF.

**Figure 9:**
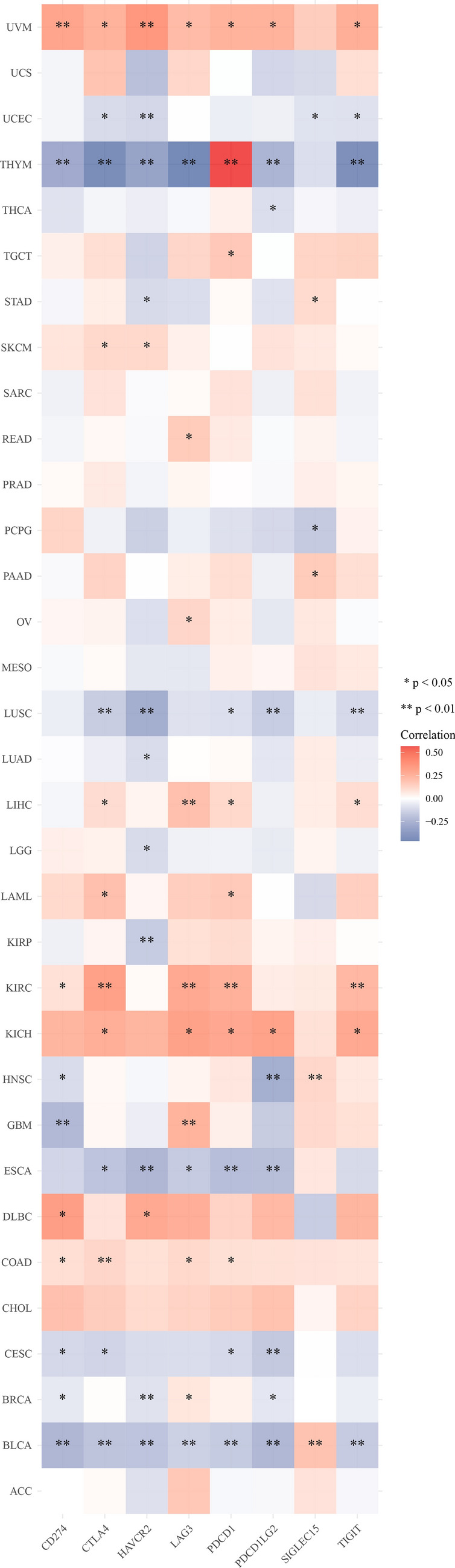
Expression of *TLX2* in pan cancer was associated with immune checkpoint genes. *P < 0.05, **P < 0.01.

As shown in Fig. 9, *TLX2* was positively or negatively correlated with the expression of immune checkpoint genes in 6 tumor types (BLCA, BRCA, GBM, HNSC, STAD, and THYM); *TLX2* expression was negatively correlated with the expression of immune checkpoint genes in 9 tumor types (CESC, ESCA, KIRP, LGG, LUAD, LUSC, PCPG, THCA, and UCEC); *TLX2* expression was positively correlated with the expression of immune checkpoint genes in 12 tumors, including (COAD, DLBC, KICH, KIRC, LAML, LIHC, OC, PAAD, READ, SKCM, TGCT, and UVM). These results suggested that *TLX2* may affect the immune response of tumors by regulating these immune checkpoint genes.

### TLX2 related pathways in pan cancer

**Figure 10:**
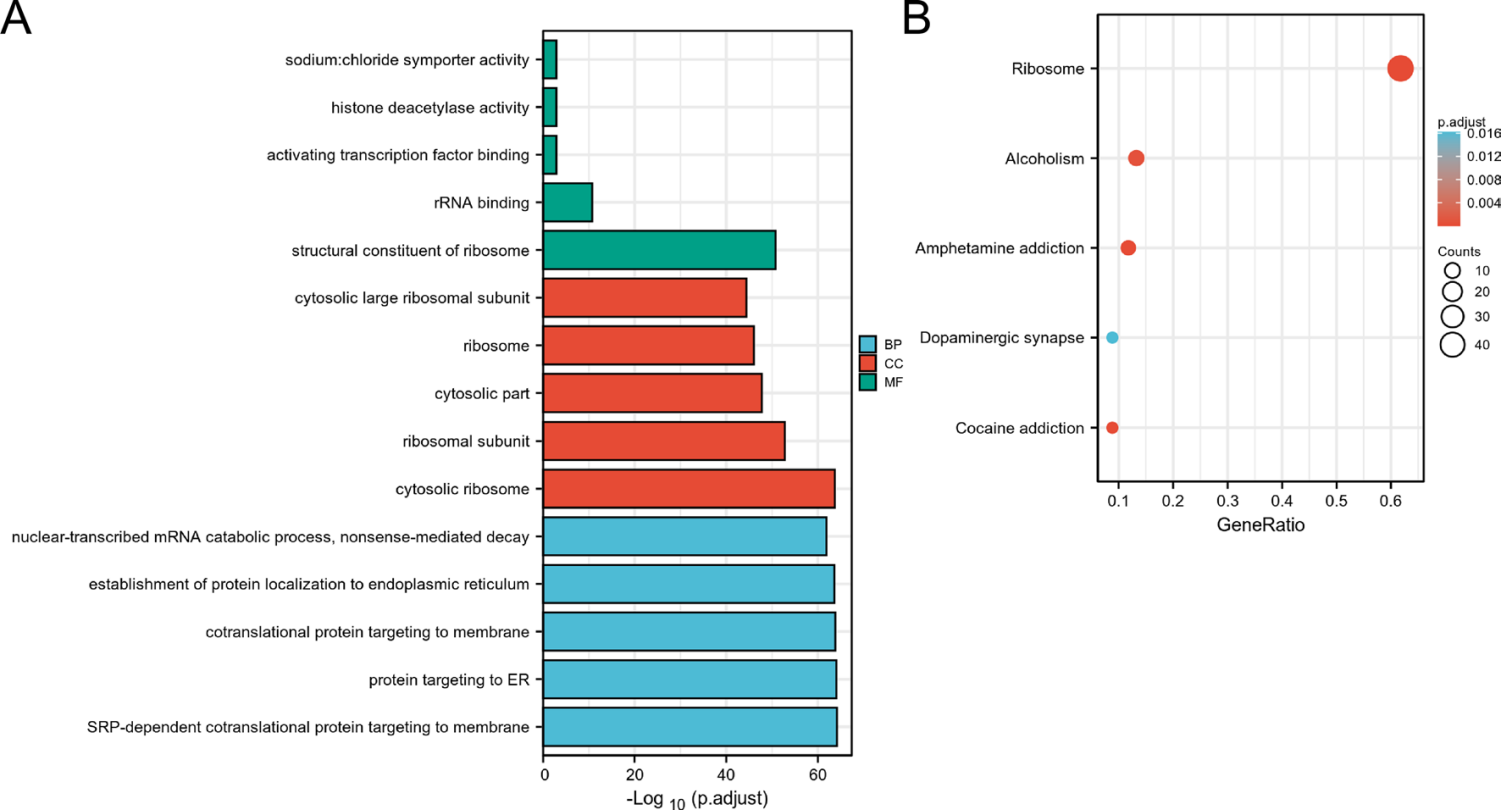
Enrichment analysis of *TLX2* and its co-expressed genes in pan cancer. (**A**) GO analysis. (**B**) KEGG analysis.

We used GEPIA2 to obtain the top 100 genes similar to *TLX2* and the STRING tool to obtain 55 genes that interacted with *TLX2*. We performed enrichment analysis on these 155 genes. The results showed 5 significantly related biology processes, including SRP-dependent cotranslational protein targeting to membrane, protein targeting to ER, cotranslational protein targeting to membrane, establishment of protein localization to endoplasmic reticulum, and nuclear-transcribed mRNA catabolic process, nonsense-mediated decay (Fig. 10A). The results showed five significantly related cell components, including cytosolic ribosome, ribosomal subunit, cytosolic part, ribosome, and cytosolic large ribosomal subunit (Fig. 10A). The results showed five significantly related molecular functions, including structural constituent of ribosome, rRNA binding, activating transcription factor binding, histone deacetylase activity, and sodium: chloride symporter activity (Fig. 10A). As illustrated in Fig. 10B, five pathways, including ribosomes, amphetamine addiction, cocaine addiction, alcoholism, and dopaminergic synapses, may be the main pathways through which *TLX2* mediates tumorigenesis and progression.

### Drug sensitivity analysis of TLX2

As shown in Table 1, *TLX2* expression positively correlated with the drug sensitivity of rutacridone epoxide, vicenistatin, n8-actinomycin d, calcein am, and thymophthalein, and negatively correlated with the drug sensitivity of Y-39983, CX-5461, VNLG/124, Navitoclax, and indole-2,3-dione, 3-[(o-nitrophenyl) hydrazone]. These suggested that *TLX2* may be associated with resistance to certain chemotherapeutic agents.

**Table 1:** Drug sensitivity analysis of *TLX2*.

Compound	Source	Spearman rho	Spearman.fdr	P value
Rutacridone epoxide	CellMiner	0.472	0.021	0.00014
Vicenistatin	CellMiner	0.420	0.022	0.00085
n8-Actinomycin d	CellMiner	0.400	0.040	0.0015
Calcein am	CellMiner	0.394	0.027	0.0018
Thymophthalein	CellMiner	0.377	0.028	0.003
Y-39983	GDSC	− 0.162	< 0.001	< 0.001
CX-5461	GDSC	− 0.164	< 0.001	< 0.001
VNLG/124	GDSC	− 0.167	< 0.001	< 0.001
Navitoclax	GDSC	− 0.178	< 0.001	< 0.001
Indole-2,3-dione, 3-[(o-nitrophenyl)hydrazone]	CellMiner	− 0.407	0.029	0.0013

### Expression of TLX2 in single cells correlated with cancer function

**Figure 11:**
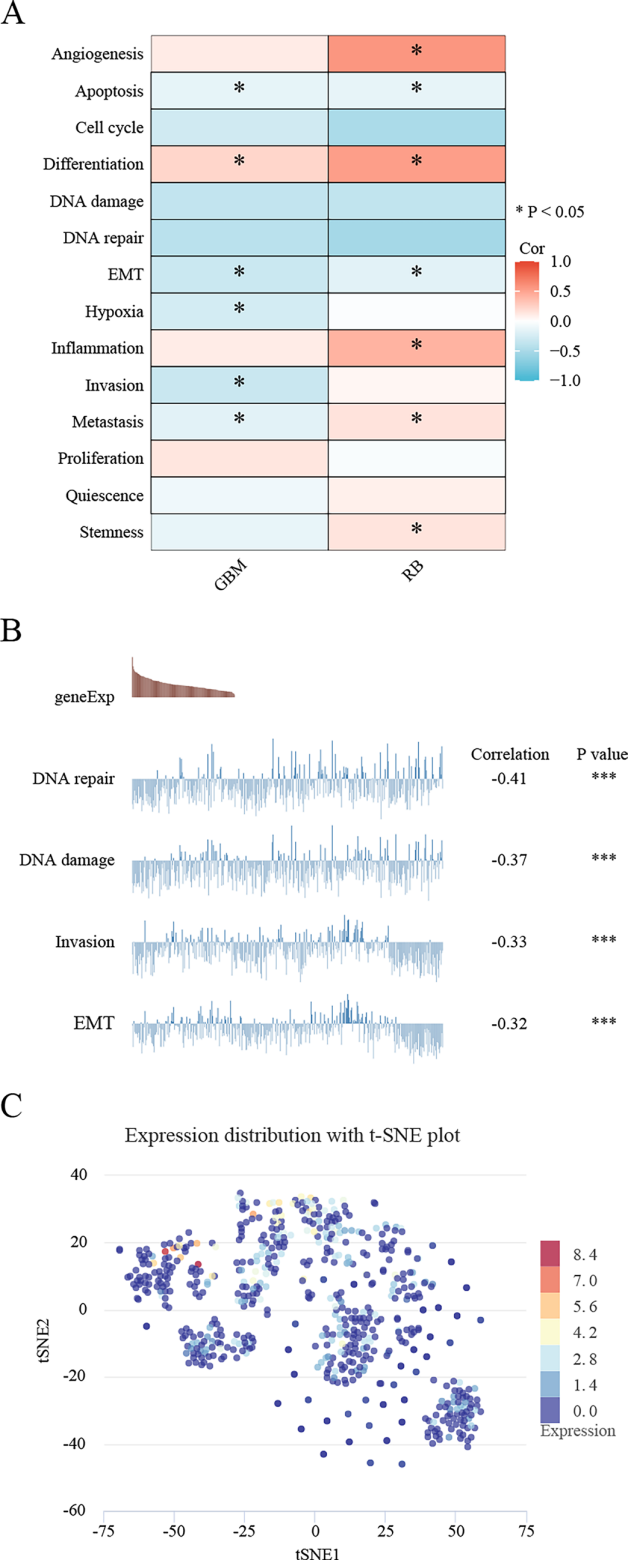
Expression pattern of *TLX2* and its correlation with tumor functional status (single cell sequencing). (**A**) Correlation between *TLX2* expression and different tumor functional states (*P < 0.05). (**B**) Correlation between *TLX2* expression and different functional status in GBM (*P < 0.05). (**C**) Expression of *TLX2* in GBM monocytes (T-SNE plots).

As shown in Fig. 11A, *TLX2* expression in GBM was positively correlated with differentiation and negatively correlated with apoptosis, cell cycle, DNA damage, DNA repair, EMT, hypoxia, invasion, and metastasis. *TLX2* expression in RB was positively correlated with angiogenesis, differentiation, inflammation, metastasis, and stemness and negatively correlated with apoptosis, cell cycle, DNA damage, DNA repair, and EMT. As shown in Fig. 11B, the results showed the relationship between *TLX2* expression and DNA repair, TLX2 expression and DNA damage, *TLX2* expression and invasion, and *TLX2* expression and EMT in GBM. T-SNE plots showed the expression profile of *TLX2* in GBM single cells (Fig. 11C). These results suggest that *TLX2* may play a role in the development of cancer progression.

### CeRNA network of TLX2

**Figure 12:**
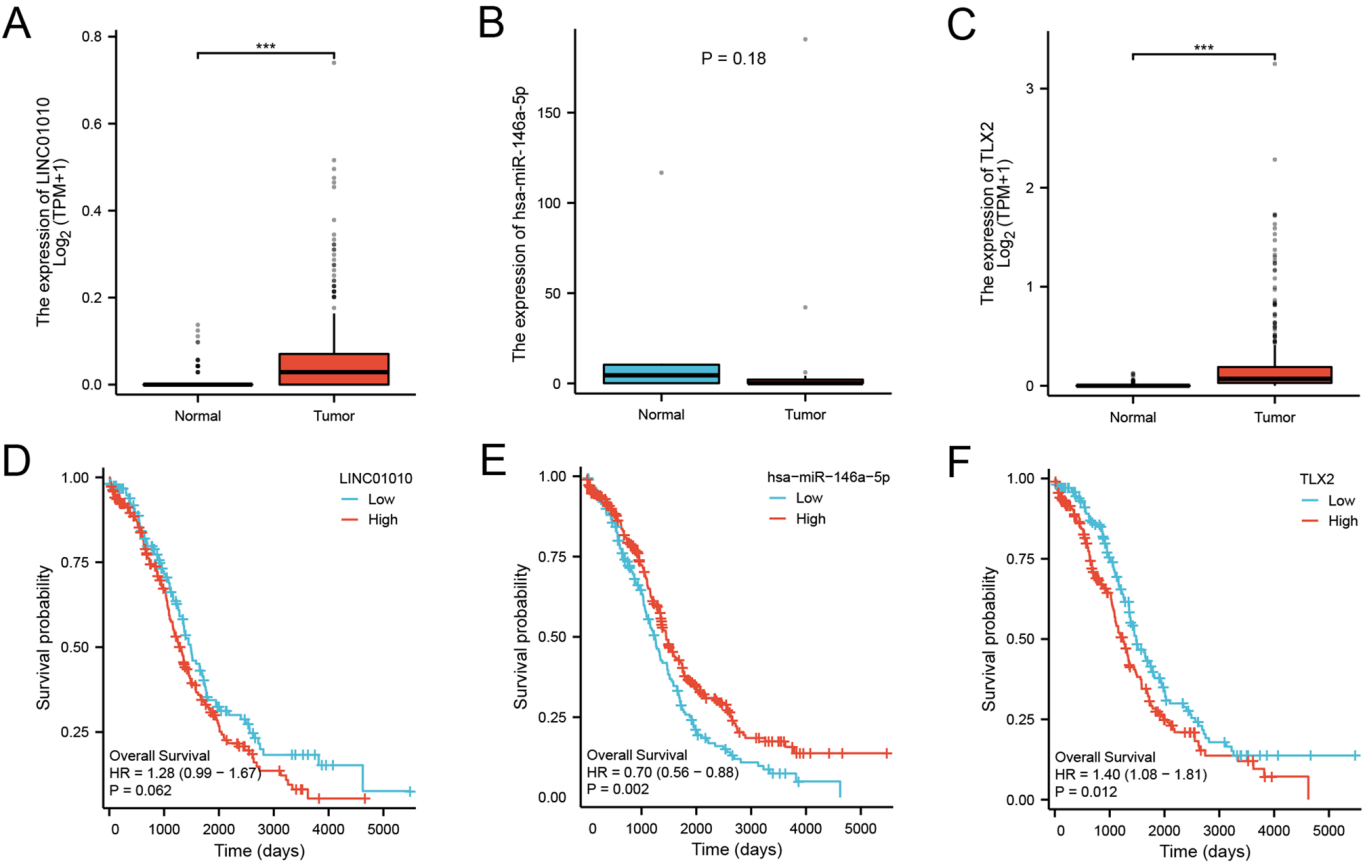
Differential expression of *TLX2*-mediated ceRNA network in OC and correlation with prognosis. (**A**) Differential expression of *LINC01010* in OC tumor tissues and normal tissues. (**B**) Differential expression of miR-146a-5p in OC tumor tissues and normal tissues. (**C**) Differential expression of *TLX2* in OC tumor tissues and normal tissues. (**D**) Prognostic value of *LINC01010* in OC. (**E**) Prognostic value of miR-146a-5p in OC. (**F**) Prognostic value of *TLX2* in OC.

We next examined the possible regulatory network of *TLX2* in OC. The expression of *LINC01010* was significantly upregulated in OC compared to normal tissues (Fig. 12A). The expression of miR-146a-5p was downregulated in OC compared with normal tissues (Fig. 12B). The expression of *TLX2* was significantly upregulated in OC compared to normal tissues (Fig. 12C). High expression of *LINC01010* in OC suggested a poorer prognosis (Fig. 12D). Low expression of miR-146a-5p in OC suggested a poorer prognosis (Fig. 12E). High expression of *TLX2* in OC suggested a poorer prognosis (Fig. 12F).

### Validation of TLX2 expression in cell lines

**Figure 13:**
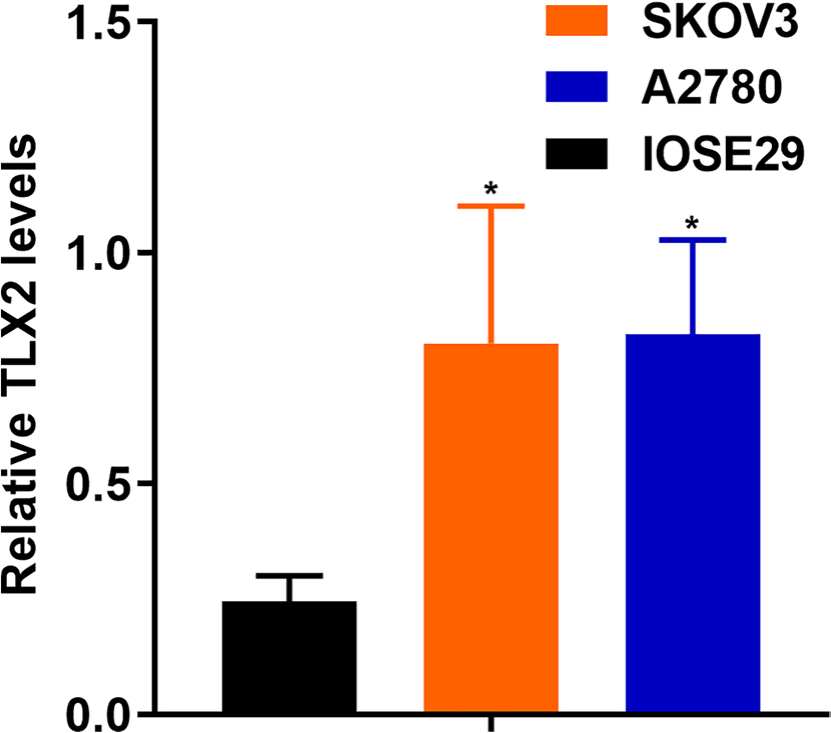
Expression of *TLX2* in human normal ovarian epithelial cells IOSE29, ovarian cancer cell lines SKOV3 and A2780.

As shown in Fig. 13, *TLX2* expression was significantly upregulated in SKOV3 compared to OSE29 (0.803 ± 0.298 vs. 0.246 ± 0.055, P = 0.0311), and *TLX2* expression was significantly upregulated in A2780 compared to OSE29 (0.824 ± 0.204 vs. 0.246 ± 0.055, P = 0.0268). These results suggest that *TLX2* expression is significantly upregulated in OC cell lines.

## Discussion

Pan cancer analysis provides insight into the molecular expression and genomic alterations of various cancers and helps to provide effective biomarkers for cancer diagnosis and treatment. TCGA includes data from 33 tumors and facilitates the discovery of differential expression of molecules and genomic alterations at the pan cancer level using multi-omics data analysis^33,34^.

Endogenous *TLX2* gene expression occurs in many non-neuroblastoma tissues, including the heart, bladder, brain, adrenal glands, and intestine^32^. However, there is a paucity of research on *TLX2* in other cancer types. Therefore, in this study, we explored the possible role of *TLX2* in pan cancer. In the present study, we found that *TLX2* was aberrantly expressed in pan cancer. *TLX2* was significantly upregulated in OC cell lines.

Aberrant abnormalities of TLX2 correlated with prognosis in LUSC^9^. Aberrant expression of *TLX2* was associated with prognosis in uterine sarcoma^10^. In this study, we investigated the correlation between *TLX2* expression and prognosis of pan cancer. Aberrant expression of *TLX2* was significantly associated with OS, PFS, and DSS in some tumors, including ACC, COAD, KIRC, LUSC, OC, READ, SARC, SKCM, and UCS. These suggested that *TLX2* may be a promising prognostic marker in pan cancer.

However, there are no relevant studies on *TLX2* gene alterations in human cancers. Thus, it was decided to use the cBioPortal database to discover that amplification is the greatest frequency of *TLX2* alterations in pan cancer. In pan cancer, *NXF2B*, *MSLNL*, *PCGF1*, *INO80B-WBP1*, *LBX2-AS1*, *MRPL53*, *LBX2*, *TTC31*, *WDR54* and *WBP1* were co-altered in the *TLX2*-altered group. PFS in the *TLX2* altered group suggested a worse prognosis in OC.

In the realm of scientific research, it has conventionally been established that intensive promoter DNA methylation is closely linked to the repression of transcription^35^. Notably, distinct modifications in the DNA methylome have been observed in the oncogenesis of various types of cancer^36,37^. In this study, we found that TLX2 presented high methylation levels and expression levels in 12 tumors (BLCA, BRCA, CESC, CHOL, ESCA, HNSC, KIRC, KIRP, LIHC, LUAD, LUSC, PAAD, PRAD, THCA, and UCEC) and high methylation levels and low expression levels in 2 tumors (COAD and READ). The TERT promoter undergoes DNA hypermethylation, resulting in an upregulation of TERT expression in cancer^38^. AML exhibits elevated levels of methylation and expression of the AWT1 gene^39^. The FOXA2 gene demonstrates heightened methylation and expression during the development of endoderm^40^. DNA hypermethylation amplifies the expression of telomerase reverse transcriptase in human induced pluripotent stem cells^41^. Our discoveries unveil a previously unknown role of DNA methylation in regulating TLX2 gene expression.

In this study, we found that *TLX2* expression correlated with stage, MSI, and TMB. High expression of *TLX2* in four tumors was significantly associated with tumor stage. Expression of *TLX2* was significantly associated with MSI in seven cancer types and TMB in five cancer types. Expression of *TLX2* may affect the response of cancer patients to immune checkpoint therapy, which will help us to further explore the mechanisms of *TLX2*-based immunotherapy in pan cancer.

CAF, as a major component of Tumor microenvironment (TME), plays an important role in tumorigenesis^42^. To date, the role of *TLX2* in the human immune system is unclear. In this study, we investigated whether *TLX2* in pan cancer was associated with immune infiltration and immune checkpoint genes. Our work elucidated the potential role of *TLX2* in tumor immunity.

TLX2 is an orphan homeodomain transcription factor whose expression is a transcriptional target of PHOX2B in neural crest-derived cells^32^. *TLX2* is a gene involved in pain transmission^43^. TLX2 homology domain can bind to 14-3-3eta signaling proteins^44^. In this study, we found that *TLX2* was associated with ribosomes, amphetamine addiction, cocaine addiction, alcoholism, and dopaminergic synapses in pan cancer. Ribosome biogenesis plays a crucial role in the process of cancer metastasis and resistance to treatment^45^. The administration of amphetamine may lead to modifications in immune function and cytokine expression, which are linked to the development of lymphoma^46^. The genotoxicity, oxidative stress, and inflammatory responses induced by crack cocaine are associated with the process of carcinogenesis^47^. Consumption of alcohol poses a risk factor for various major cancers^48^. Par-4, which was identified through a screening process for genes with heightened expression in prostate tumor cells undergoing apoptosis, mediates dopaminergic synaptic plasticity^49^. *TLX2* may mediate tumorigenesis and progression through these pathways.

Undoubtedly, there are no studies correlating *TLX2* with drug sensitivity or resistance. We used the RNAactDrug database to analyze and found that *TLX2* expression correlated with many drug sensitivities, including Y-39983, CX-5461, VNLG/124, Navitoclax, and indole-2,3-dione, 3-[(o-nitrophenyl) hydrazone]. *TLX2* may be associated with drug resistance.

In the present study, CancerSEA had only data for GBM and RB. We found that *TLX2* expression in GBM was significantly and positively correlated with differentiation and negatively correlated with apoptosis, cell cycle, DNA damage, DNA repair, EMT, hypoxia, invasion, and metastasis. However, the expression of *TLX2* in OC single-cell sequencing is unclear and deserves further in-depth study.

However, there were some limitations of this study. First, this study explored the clinical significance and genomic variation of *TLX2* aberrant expression in pan cancer based on public databases, and these may require real-world samples for validation in the future. Second, we explored the possible regulatory network and drug sensitivity of *TLX2* in pan cancer, and the specific mechanisms of *TLX2* in pan cancer development and progression need to be further investigated. Finally, we constructed a ceRNA network of *LINC01010*/miR-146a-5p/*TLX2* in OC, and the results of these bioinformatics analyses need some experiments for validation.

## Conclusion

*TLX2* can be a candidate diagnostic and prognostic factor in a variety of cancers. *TLX2* expression was associated with MSI, TMB, CAF infiltration, immune checkpoint genes, and drug sensitivity. The methylation levels of TLX2 exhibited high values in 17 tumors. The possible ceRNA network of *LINC01010*/miR-146a-5p/*TLX2* was constructed in OC. This study explored the role of *TLX2* in pan cancer and laid the foundation for the use of *TLX2* as a new diagnostic and prognostic molecular marker and therapeutic strategy.

### Supplementary Information

Supplementary Legends.

Supplementary Figure S1.

Supplementary Table S1.

## Data Availability

All data generated or analyzed during this study are included in this article.
