# Author Correction: Comprehensive analysis of TLX2 in pan cancer as a prognostic and immunologic biomarker and validation in ovarian cancer

**DOI:** 10.1038/s41598-023-44831-y

**Published:** 2023-10-17

**Authors:** Buze Chen, Xiaojuan Ding, Ailing Wan, Xin Qi, Xiaoman Lin, Haihong Wang, Wenyu Mu, Gang Wang, Junnian Zheng

**Affiliations:** 1 Xuzhou Medical University, Xuzhou, 221004 Jiangsu China; 2 Cancer Institute, Xuzhou Medical University, No. 209 Tongshan Road, Yunlong District, Xuzhou, 221004 Jiangsu China; 3 Department of Gynecology, The Affiliated Hospital of Xuzhou Medical University, No. 99 West Huaihai Road, Quanshan District, Xuzhou, 221002 Jiangsu China; 4 Center of Clinical Oncology, The Affiliated Hospital of Xuzhou Medical University, No. 99 West Huaihai Road, Quanshan District, Xuzhou, 221002 Jiangsu China; 5 Jiangsu Center for the Collaboration and Innovation of Cancer Biotherapy, Xuzhou Medical University, Xuzhou, 221004 Jiangsu China

Correction to: *Scientific Reports* 10.1038/s41598-023-42171-5, published online 27 September 2023

The original version of this Article contained errors in Figure 12, where panels B and C as well as panels E and F were reversed. The original Figure 12 and accompanying legend appear below.

**Figure 12:**
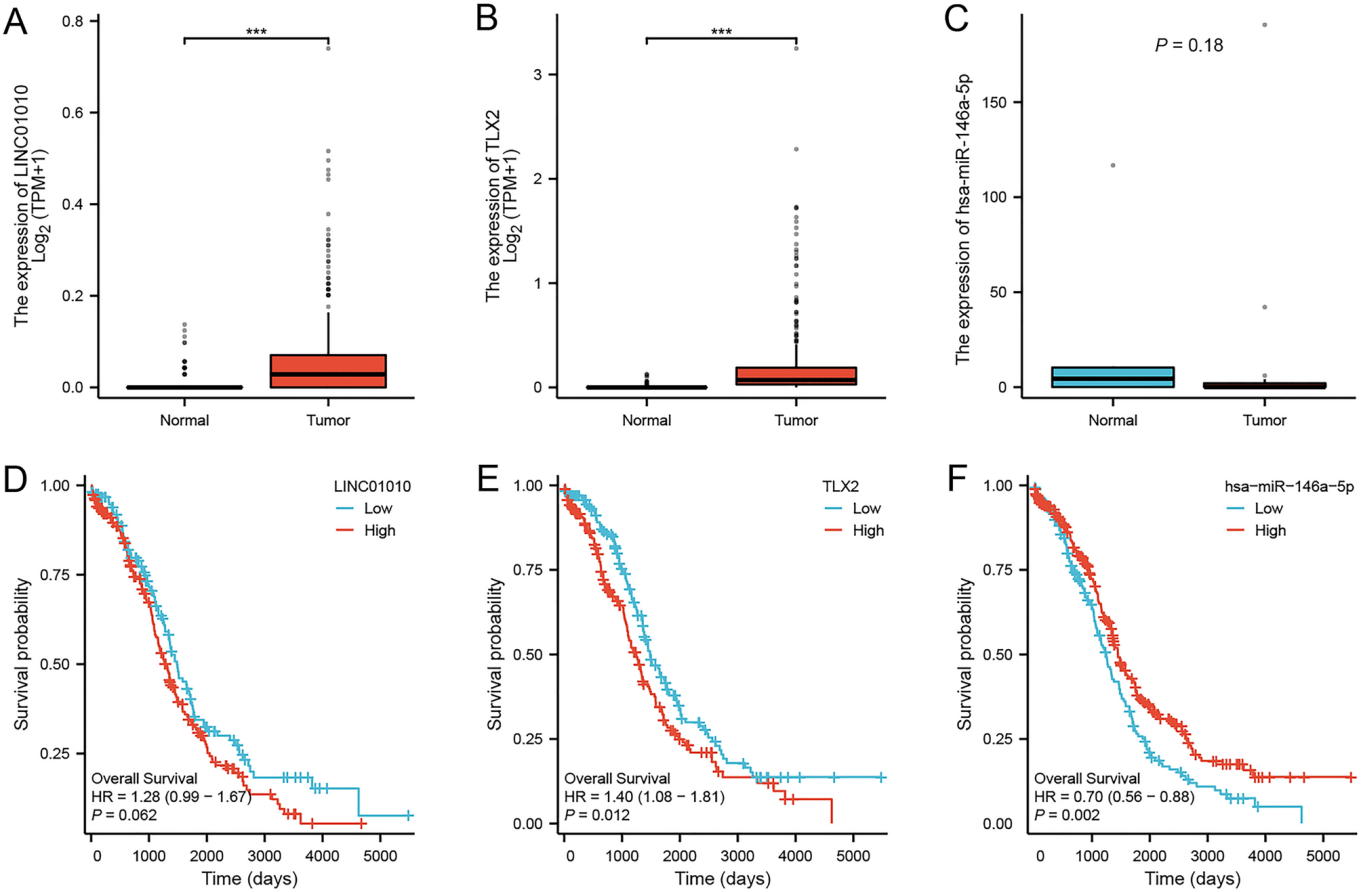
Differential expression of *TLX2*-mediated ceRNA network in OC and correlation with prognosis. (**A**) Differential expression of *LINC01010* in OC tumor tissues and normal tissues. (**B**) Differential expression of miR-146a-5p in OC tumor tissues and normal tissues. (**C**) Differential expression of *TLX2* in OC tumor tissues and normal tissues. (**D**) Prognostic value of *LINC01010* in OC. (**E**) Prognostic value of miR-146a-5p in OC. (**F**) Prognostic value of *TLX2* in OC.

The original Article has been corrected.

