# Supplementary figures and images for: Comprehensive analysis of TLX2 in pan cancer as a prognostic and immunologic biomarker and validation in ovarian cancer
Source: Sci Rep. 2023 Sep 27;13:16244. doi: 10.1038/s41598-023-42171-5 (PMC10533500; doi:10.1038/s41598-023-42171-5)

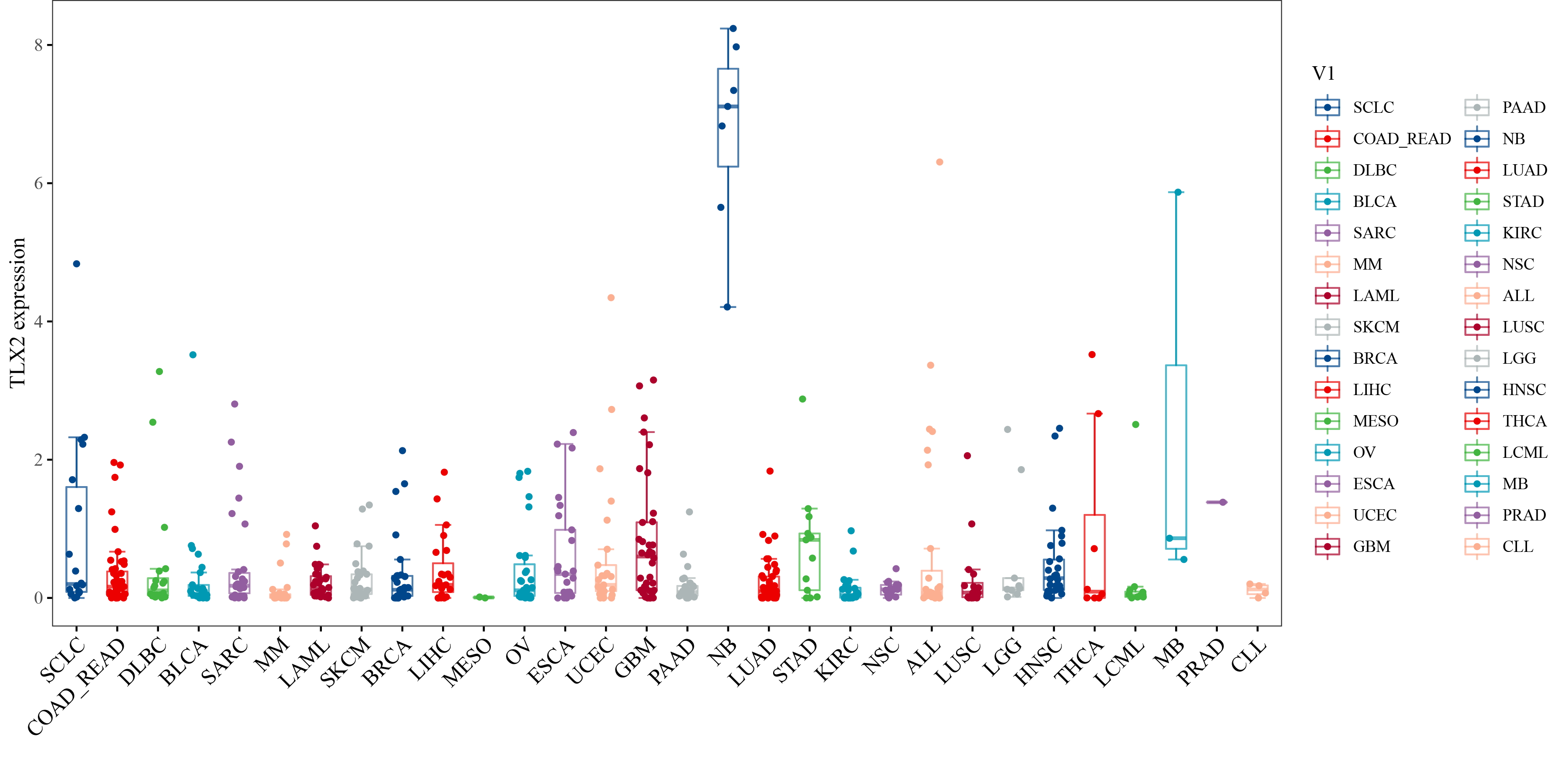

Supplement: Supplementary file 2 — Supplementary Figure S1. [file 41598_2023_42171_MOESM2_ESM.tif]
